# Octocoral Tissue Provides Protection from Declining Oceanic pH

**DOI:** 10.1371/journal.pone.0091553

**Published:** 2014-04-07

**Authors:** Yasmin Gabay, Maoz Fine, Zahava Barkay, Yehuda Benayahu

**Affiliations:** 1 Department of Zoology, Tel-Aviv University, Tel-Aviv, Israel; 2 The Interuniversity Institute for Marine Science, Eilat, Israel; 3 The Mina and Everard Goodman Faculty of Life Sciences, Bar-Ilan University, Ramat-Gan, Israel; Heriot-Watt University, United Kingdom

## Abstract

Increase in anthropogenic *p*CO_2_ alters seawater chemistry and could lead to reduced calcification or skeleton dissolution of calcifiers and thereby weaken coral-reef structure. Studies have suggested that the complex and diverse responses in stony coral growth and calcification, as a result of elevated *p*CO_2_, can be explained by the extent to which their soft tissues cover the underlying skeleton. This study compared the effects of decreased pH on the microstructural features of both in hospite (within the colony) and isolated sclerites (in the absence of tissue protection) of the zooxanthellate reef-dwelling octocoral *Ovabunda macrospiculata*. Colonies and isolated sclerites were maintained under normal (8.2) and reduced (7.6 and 7.3) pH conditions for up to 42 days. Both in hospite and isolated sclerites were then examined under SEM and ESEM microscopy in order to detect any microstructural changes. No differences were found in the microstructure of the in hospite sclerites between the control and the pH treatments. In stark contrast, the isolated sclerites revealed dissolution damage related to the acidity of the water. These findings suggest a protective role of the octocoral tissue against adverse pH conditions, thus maintaining them unharmed at high *p*CO_2_. In light of the competition for space with the less resilient reef calcifiers, octocorals may thus have a significant advantage under greater than normal acidic conditions.

## Introduction

Ocean acidification, the continuing global decline in oceanic pH resulting from rising atmospheric carbon dioxide (CO_2_), also reduces carbonate ion concentration ([CO_3_^2−^]) and saturation state (Ω), which are essential components of the CaCO_3_ mineral from which marine calcifiers build their shells and skeletons [1]–[3]. The effect of ocean acidification on coral reefs, in particular on stony corals (Scleractinia), the main reef framework-builders, has been extensively studied. Several species exhibited a significantly decreased skeletal growth under high CO_2_ concentrations, including *Stylophora pistillata* [4]–[5], *Acropora* sp. [6]–[8], *Montipora capitata* and *Porites compressa* [9]–[10], *Oculina patagonica* and *Madracis pharencis* [11], as well as *M. auretenra* [12]. A species-specific response of stony corals to lower pH has often been explained by the extent to which the living tissue covers the underlying skeleton. For example, under lower pH *Cladocora caespitosa*, which features large areas of exposed skeleton, underwent skeletal dissolution, while the fully tissue-covered *Balanophyllia europea* did not exhibit any signs of dissolution [13]. Rodolfo-Metalpa et al. [14] highlighted the role played by an external organic layer as a protective barrier against the harmful effects of lower pH, and demonstrated that dead colonies of the bryozoan *Myriapora truncata* and mollusk shells underwent dissolution at high CO_2_ levels, as opposed to the respective live specimens, which maintained the same net calcification rate as that occurring under normal conditions.

Octocorals of the order Alcyonacea are widespread throughout the tropical Indo-Pacific region and considered the second most important benthic component after stony corals on many of these reefs [15]–[16]. Most octocorals lack a massive hard skeleton for support; instead, they generate hydrostatic pressure by means of internal calcite skeletal elements, termed sclerites. The shape, size, and even coloration of sclerites vary widely among taxa and, therefore, have been used for taxonomic identification [16]. The sclerites are found in varying densities and embedded in the fleshy coenenchyme of the colony [17]. Unlike stony corals, which deposit calcium carbonate as aragonite crystals, octocoral sclerites are made of either separate or fused polycrystalline aggregates of high magnesium calcite [18]–[19]. Gabay et al. [20] demonstrated that under more acidic conditions than normal (pH 7.6 and 7.3), no change took place in the biological features of several octocoral species. Protein concentration, polyp weight, density of zooxanthellae, and their chlorophyll concentration per cell, as well as polyp pulsation rate, did not vary between the treatments and control. Octocorals may also serve as reef builders, being able to cement sclerites and consolidate them at the colony base into spiculite [17]. Intrigued by the ecological function of octocorals on coral reefs, we sought to determine the response of sclerites to a scenario of increased *p*CO_2_, as well as to determine the possible protective function of the tissue. The present study thus compares the effects of decreased pH on the microstructural features of both in hospite (within the colony) and isolated sclerites (i.e. in the absence of tissue protection) of the reef-dwelling octocoral *Ovabunda macrospiculata* (family Xeniidae).

## Materials and Methods

### Animals and experimental system

The study was conducted at the Interuniversity Institute (IUI) for Marine Sciences in Eilat. Colonies of the octocoral *O. macrospiculata* were collected by SCUBA (2009–2010) from the reef across from the IUI at a depth of 8–12 m (collection of animals complied with a permit issued by the Israel Nature and National Parks Protection Authority). Following two weeks of acclimation in a flow-through seawater table, the colonies were transferred to the experimental system. The system comprised three water tables with two pH treatments: pH 7.6 and 7.3 (*p*CO_2_ = 1917 and 3898 µatm, respectively) and a control pH 8.2 (*p*CO_2_ = 387 µatm), with the latter corresponding to the ambient Eilat seawater [21]. Table 1 presents the seawater-chemistry. The experimental pH values were determined following preliminary experiments that had revealed only a minor response of the octocorals to pH 7.9. We therefore selected a lower pH of 7.3, which is twice as high, in terms of *p*CO_2_, as that needed to reach pH 7.9. In Gabay et al. [20] we demonstrated that octocorals might be able to cope with pH levels lower than predicted for the end of this century. Therefore, in the current study we adhered to the previous pH levels to examine their effect on sclerite structure. The diurnal pH fluctuations in the Eilat reefs are in the order of 0.1 units, similar to that recorded in the pH system used in the current study [21]. Seawater was pumped into the experimental system from the reef (30 m), into three 1,000 liter tanks. The pH values (i.e., 7.3, 7.6 and 8.2) were achieved by bubbling pure CO_2_ gas stored in a cylinder through seawater to reach the desired pH. A pH electrode (S-200C, Sensorex, CA, USA) was located in each tank and connected to a pH controller (Aquastar, IKS ComputerSystem GmbH, Karlsbad, Germany) to control the gas flow. A pH deviation within the tank triggered the computer to activate a solenoid to either increase or decrease the flow of CO_2_, as necessary. The pH data were recorded using monitoring software (Timo, Matuta, Germany). The pH in the aquarium was monitored and logged every two minutes and the electrodes were calibrated once a week, or when discrepancies were detected. The seawater temperature was maintained at ∼25°C, using a combination of an array of 150 W BluClima aquarium heaters (Ferplast Spa, Vicenza, Italy) and an air-conditioner in the wet-laboratory. Water motion in the tanks was maintained by power heads (Atman, At-301, China). The octocorals were positioned under 400 W metal halide lamps, supplying ∼200 µmol quanta m^−1^ s^−1^, under a 10 h light: 14 h dark regime. The zooxanthellate xeniids are known as non-plankton feeding colonies [22] and, therefore, no food was added to the system following collection from the reef.

**Table 1:** Carbonate chemistry parameters of treatments and control calculated from pH, total alkalinity, temperature (25°C), and salinity (40 ppm) using the program CO2SYS (Lewis & Wallace, 1998).

pH NBS	TA (µeqv kg^−1^)	DIC (µmol kg^−1^)	*p*CO_2_ (µatm)	CO_2(aq)_ (µmol kg^−1^)	HCO_3_^−^ (µmol kg^−1^)	CO_3_^2−^ (µmol kg^−1^)	Ω_arg_
8.2	2501	2122	387	10.6	1846	265	4.02
7.6	2499	2431	1917	52	2295	82	1.25
7.3	2501	2544	3898	107.1	2393	44	0.67

### Effect on sclerite microstructure

#### In hospite sclerites

For each experiment colonies of *O. macrospiculata* (n = ∼100) were randomly distributed among nine transparent PVC containers (6 L). The containers were further subdivided into two pH treatments (7.3 and 7.6) and control (8.2), each with an individual tube supplying water with respective pH and an air stone, which did not affect the water chemistry (JUN ACO-5503, Guangdong Hailea, China air pump). To examine the effect of pH on the microstructure of sclerites within living colonies, they were maintained in the experimental system for 31–42 days. On each sampling date six random polyps were removed from each colony (6–8 colonies/sampling for each treatment) and placed in filtered seawater (FSW, 0.2 µm pore-size Millipore filter). The sclerites were obtained by dissolving the tissue with 10% sodium hypochlorite followed by repeated rinsing in double-distilled water (DDW) in order to remove bleach and debris, followed by a wash (2–3 min) with 95% ethanol which prevents sclerite adherence [23]. The ethanol was then discarded and the sclerites were maintained overnight at room temperature to dry. Two experiments were performed: April–May 2009 (42 days), and August–September 2010 (31 days), with sclerite samples prepared for SEM examination on the last day of each incubation (see below).

#### Isolated sclerites

To examine the effects of pH on isolated sclerites, two experiments were conducted: in August–September 2010 (31 days) and December 2010 (18 days). Living colonies (n = 18) were collected and groups of 15–60 polyps were immediately removed from each colony, and their sclerites were obtained as described above. The sclerites of each colony were placed separately in darkened 25 ml vials, covered with black masking tape (n = 6 vials for each pH treatment and control) and placed in three transparent 6 L PVC containers (n = 1 container for each pH value), individually supplied with water of the respective pH (7.3, 7.6 and 8.2). The containers were covered with aluminum foil to prevent any exposure to light that might cause the development of photosynthetic organisms and thus could affect the pH. The pH of the water in the vials was monitored twice daily using a pH electrode and the sclerites were then gently stirred to ensure full exposure to the surrounding water. For each experiment samples of sclerites (∼200 µl each) were retrieved from the vials of each pH value at time 0 and upon termination (day 31 and 18, see above), and placed in 2 ml tubes with FSW (0.2 µm) for microstructural studies. Analyzed sclerites were not returned to the experimental system.

#### Microstructure of sclerites

To examine the microstructure of the sclerites, each sample from the above experiments was separately filtered through a 0.45 µm pore-size Millipore filter. The filters were dried at room temperature, glued to stubs and gold-coated [24]. Each stub contained several hundred sclerites, viewed under a JEOL 840A SEM at 15 kV and a Quanta 200 FEG (Field Emission Gun) ESEM at 5–20 kV. In order also to view samples as closely as possible to their natural state, representative samples from the pH treatments and the control were not coated with the conducting layer of gold, but instead we used the low vacuum ESEM imaging mode. The non-conducting sclerite-samples were imaged with the secondary electron large-field detector (LFD) at low vacuum mode of 70 Pa, which provided electric charge neutralization. The microstructure of gold-coated and uncoated sclerites was compared in order to exclude a possible artifact due to the coating procedure. For sclerites of the different examination dates at least 20 represented images were photographed at each time point for further analysis (see also below).

#### Scoring of damage

SEM and ESEM micrographs of in hospite and isolated sclerites were used to determine the possible effect of pH on the sclerite-microstructure, adopting a 0–4 visual ranking-scale for the percentage of dissolution-damage featured by the microscleres comprising the sclerites (n = 18–246 sclerites/colony, 3 colonies/treatment at each time point for each experiment). Rank 0 indicated no observed dissolution-damage, 1: 1–20% damage of the sclerite surface, 2: 20–40%, 3: 40–60% and 4: >60%. The percentage dissolution-damage of the sclerites was calculated using the following equation:
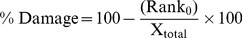
(1)and the relative average of each rank was calculated according to the equation
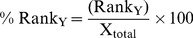
(2)where Rank_0_ refers to the number of sclerites featuring no damage; Rank_Y_ refers to each rank (0–4), and X_total_ is the total number of sclerites examined in each sample. Thus, for each treatment per date the cumulative percentage rank is 100. Analysis of variance (ANOVA) was performed on the data using SPSS 15.0 and STATISTICA 8. Log transformation was conducted on part of the data in order to achieve normal distribution (see Results). Results are expressed as mean ± standard deviation (STDEV).

## Results

### In hospite sclerites

**Figure 1:**
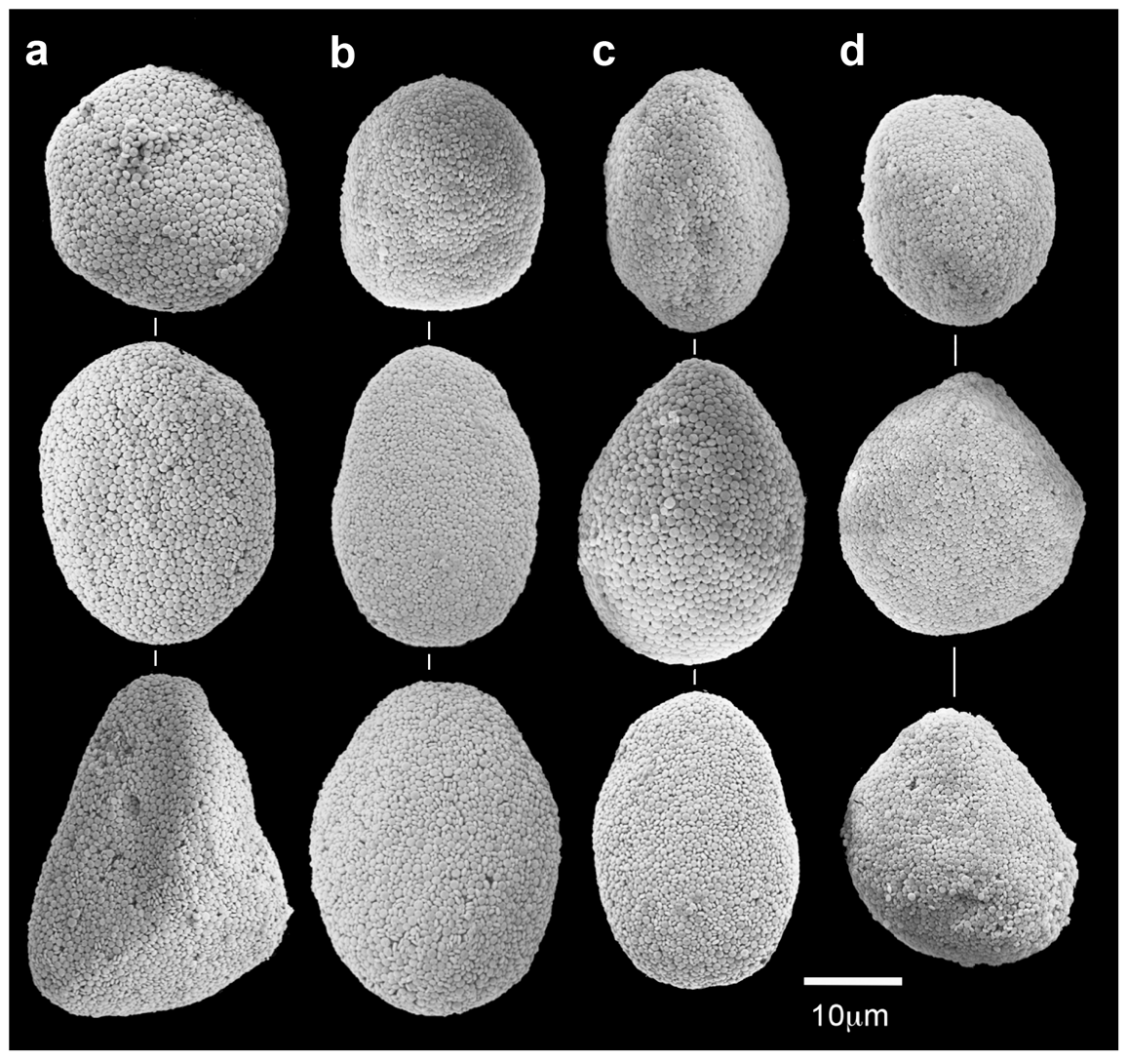
*Ovabunda macrospiculata*: SEM images of in hospite sclerites (August–September 2010 experiment). a. Day 0. Day 31: b. pH 8.2, c. pH 7.6, d. pH 7.3.

The SEM micrographs revealed that in hospite sclerites of *O. macrospiculata* that were maintained in the experimental system did not undergo any microstructural changes in both experiments (up to 42 days). Figure 1a presents images of sclerites from colonies upon collection from the reef; Fig. 1b from colonies maintained in the experimental system under pH 8.2; Fig. 1c under pH 7.6; and Fig. 1d under pH 7.3 (day 31, August–September 2010 experiment). All images of Fig. 1 feature the normal diagnostic spheroid sclerites of the genus *Ovabunda* in general and *O. macrospiculata* in particular, distinctly composed of juxtaposed corpuscular-shaped microscleres [16], [24].

### Isolated sclerites

**Figure 2:**
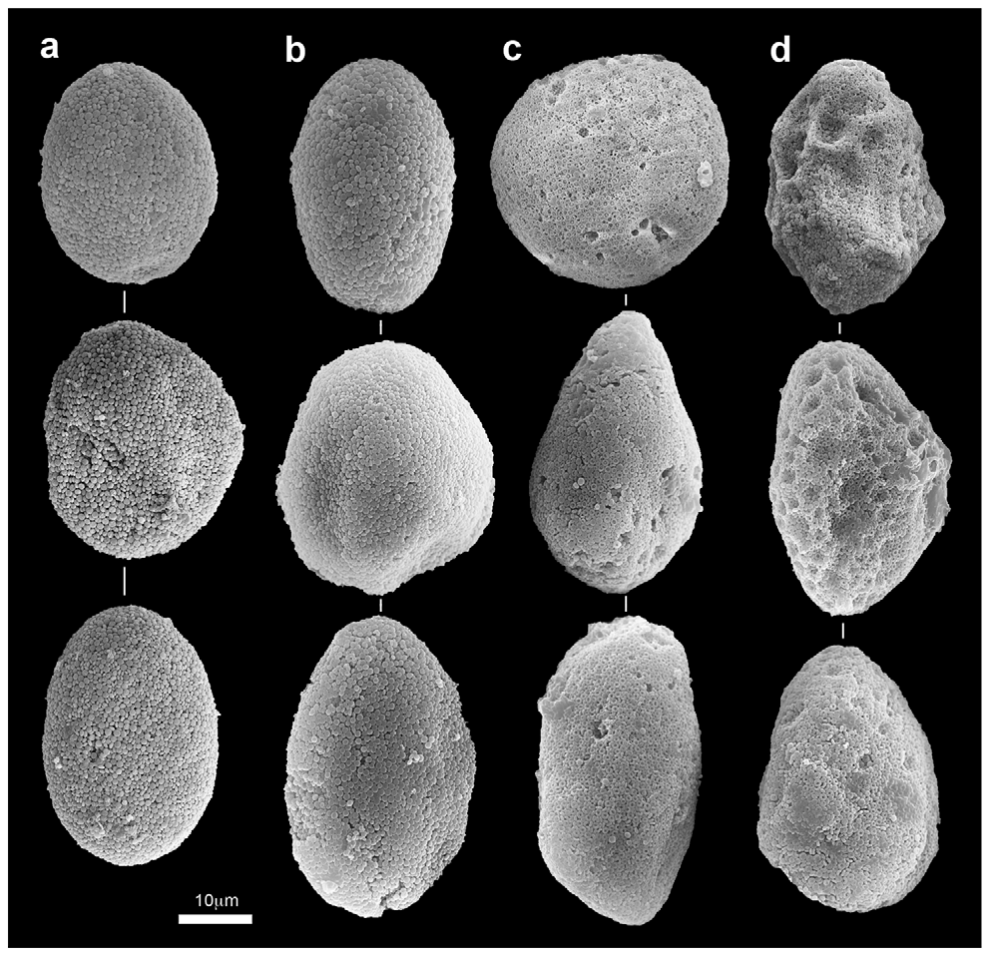
*Ovabunda macrospiculata*: SEM images of isolated sclerites (August–September and December 2010 experiments). a. Day 0. Day 18: b. pH 8.2, c. pH 7.6, d. pH 7.3.

**Figure 3:**
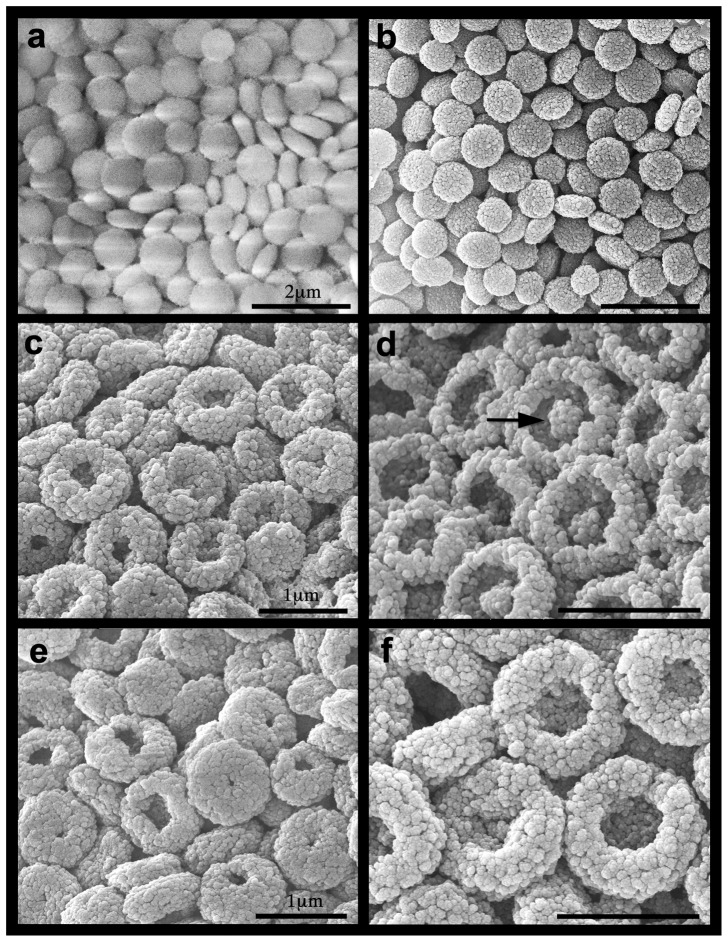
*Ovabunda macrospiculata*: ESEM images of isolated sclerites (December 2010 experiment). Day 31: a. pH 8.2 gold coated, b. uncoated, c, d. pH 7.6, e, f. pH 7.3. Arrow at d indicates dissolved circumferential zone. Scale at a applies also to b, scale at c applies also to d–f.

Isolated sclerites under pH 8.2 maintained their normal features, whereas those at pH 7.6 and 7.3 underwent microstructural changes over time. Fig. 2a presents SEM micrographs of isolated sclerites from the two experiments upon their introduction into the experimental system; and Fig. 2b on day 31 under pH 8.2, both featuring the normal *Ovabunda* spheroid sclerites. Under pH 7.3 and pH 7.6, the sclerites lost their typical morphology, exhibiting a progressive dissolution in the form of pits on their surface, visible even under relatively low SEM magnification (Fig. 2c, d). High ESEM magnifications further demonstrated the corrosive effect of the reduced pH treatments on the microscleres compared to the control (Fig. 3). The microstructure of both uncoated (Fig. 3a) and gold-coated (Fig. 3b) sclerites under pH 8.2 for 31 days (December 2010) exhibited the typical corpuscular-shaped microscleres. These images indicate that their granular appearance following the latter treatment (Fig, 3b) is due to the gold coating, as similarly found at all time-points. Since the coated sclerites yielded sharp images they were further applied and presented in the current study. The pH 7.6 and 7.3 treatments led to a repetitive dissolution-pattern of the microscleres' center (Figs. 3c–f). Under pH 7.6 the magnitude of the corrosion affected the microscleres in such a manner that, on day 18, although their outer rim and center remained, a dissolved circumferential zone was left in-between (Fig. 3c, d). Under pH 7.3, further dissolution occurred, mostly resulting in a pit-formation at the center of the microsclere, leaving only its outer rim intact (Fig. 3e, f).

### Scoring of damage

**Figure 4:**
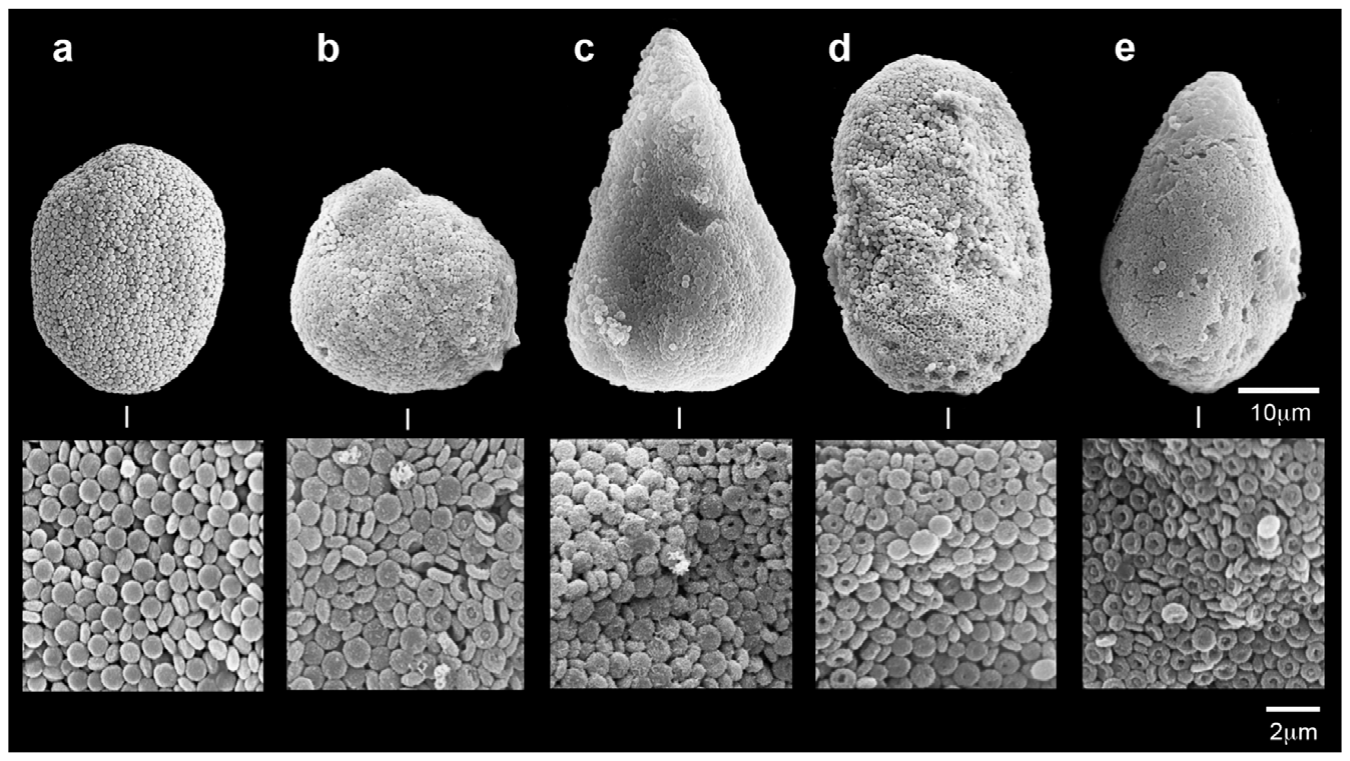
*Ovabunda macrospiculata*: Visual score of sclerite damage a. Rank 0, 0% damage, b. Rank 1, 1–20% damage, c. Rank 2, 21–40% damage, d. Rank 3, 41–60% damage, e. Rank 4, >60% damage.

**Figure 5:**
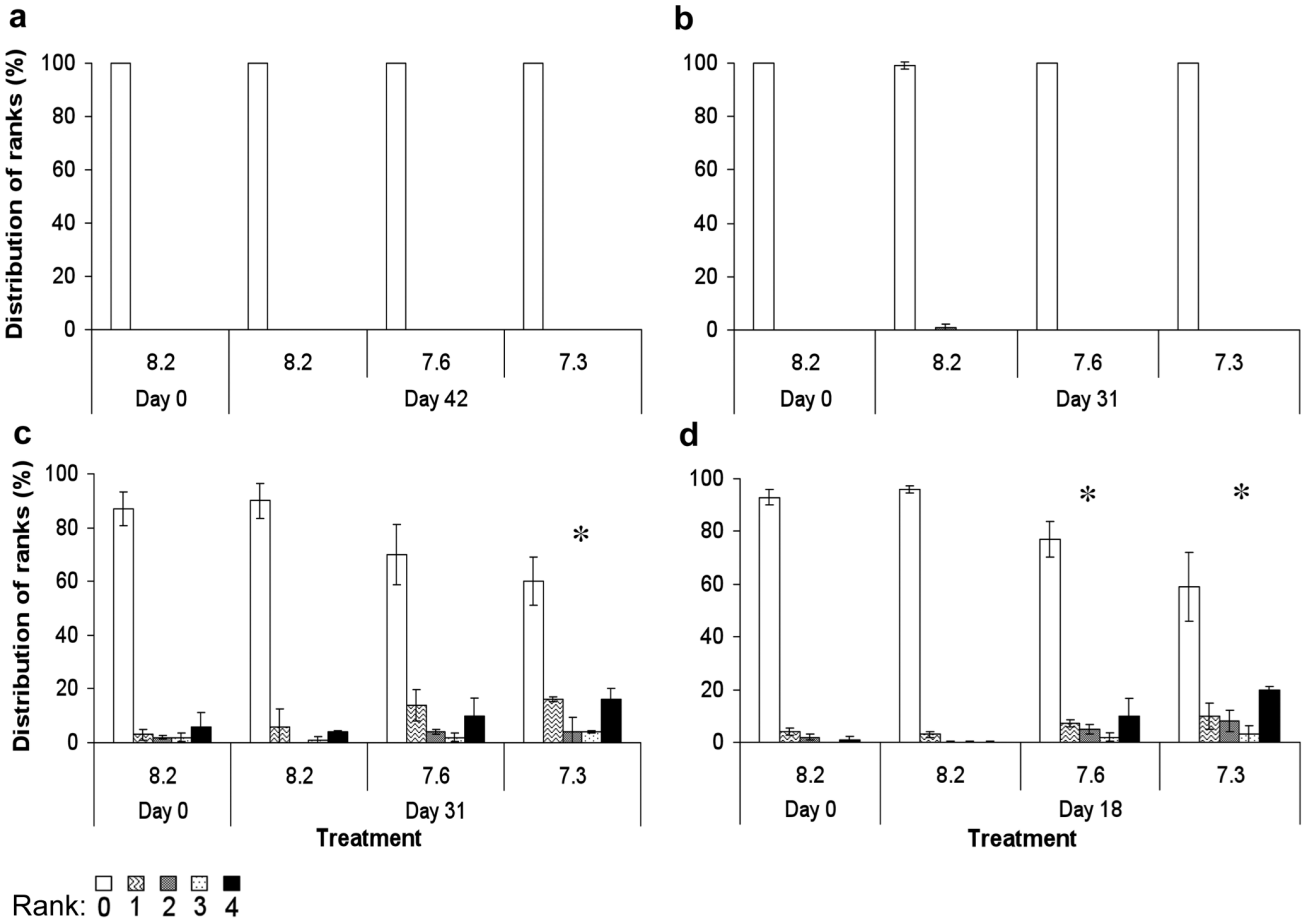
*Ovabunda macrospiculata*: Frequency distribution of damage ranking of sclerites (%, ±SD) under normal (pH 8.2) and experimental (pH 7.6, 7.3) conditions over time. a, b. in hospite sclerites (April–May 2009, and August–September 2010, respectively). c, d. isolated sclerites (August–September and December 2010, respectively). Rank 0: 0% damage, Rank 1: 1–20% damage, Rank 2: 21–40% damage, Rank 3: 41–60% damage, Rank 4: >60% damage (n = 18–246 sclerites per colony, 3 colonies per treatment at each time point for each experiment), * *p*<0.05 (One-Way ANOVA, Tukey post-hoc).

Figure 4a presents SEM images with no damage to the sclerite and its microscleres corresponding to rank 0 and Fig. 4b–e- presents progressive dissolution effects, corresponding to ranks 1–4, respectively. SEM micrographs of in hospite sclerites of April–May 2009 and August–September 2010 experiments revealed no microstructural changes (Fig. 5a, b). In the first experiment sclerites in both the control (pH 8.2, n = 272 sclerites) and the treatments (pH 7.6, n = 392; pH 7.3, n = 358 sclerites) were ranked 0 after 42 days (Fig. 5a). Similarly, in hospite sclerites of the second experiment revealed no microstructural damage (Fig. 5b). The sclerites of the control at time 0 were ranked 0 (n = 169 sclerites), as well as sclerites in the treatments (pH 7.6, n = 193; pH 7.3, n = 92 sclerites) after 31 days. At day 31 only the control featured 1% damage (n = 185 sclerites).

SEM micrographs of isolated sclerites in both August–September and December 2010 experiments revealed significant microstructural changes between the treatments and the control as demonstrated in Fig. 5c, d. In the first experiment significant differences were found between the corrosion that occurred in the control and in the treatments (following log transformation, One-Way ANOVA, *p* = 0.010, F = 10.736; Fig. 5c). A significant difference was found between sclerites in the control and in pH 7.3 (Tukey post-hoc). The sclerites of the control at both time 0 and on day 30 were ranked 1, featuring 13±6 (n = 213 sclerites) and 10±7% (n = 99 sclerites) damage, respectively. The average dissolution that took place under pH 7.6 and pH 7.3 was 30±11 (n = 101 sclerites) and 40±9% (n = 56 sclerites) respectively, both ranked 2. In the second experiment similar significant differences were found between the treatments and the control (One-Way ANOVA, *p* = 0.001, F = 24.768; Fig. 5d). A significant difference was found between sclerites in the control and in both pH treatments (pH 7.3 and 7.6) (Tukey post-hoc). The control at both time 0 and on day 18 was ranked 1, with 7±3 (n = 257 sclerites) and 4±1% (n = 564 sclerites) dissolution-damage, respectively. Under pH 8.2 the sclerites ranking 0 comprised 93±3 and 96±1% of the total examined sclerites on days 0 and 18, respectively (Fig. 5d). The average damage that occurred under pH 7.6 was 23±7% and ranked 2 (n = 271 sclerites), whereas under pH 7.3 it was 41±13% and ranked 3 (n = 251 sclerites).

## Discussion

The current study examined the effects of reduced pH on the microstructure of in hospite sclerites *vs.* isolated ones of the reef-dwelling octocoral *O. macrospiculata*. It was found that the former had remained intact (Fig. 1), while the latter had undergone a remarkable dissolution (Figs. 2–5). These findings thus suggest a possible protective role of the octocoral tissue against corrosion of in hospite sclerites under acidic conditions. Research conducted to date has revealed various effects, mostly negative, of reduced pH on external calcite-composed skeleton of marine organisms [25]. The responses of scleractinian corals to ocean acidification would seem to range from total skeleton dissolution to increased calcification rate. For example, *Oculina patagonica* [11] and primary polyps of *Favia fragum* [26] exhibited substantial skeleton dissolution under such conditions. Coccolithophores [27] and crustaceans [25] exhibited increased calcification. Rodolfo-Metalpa et al. [14] demonstrated that the skeleton of dead colonies of the bryozoan *Myriapora truncata* dissolved at pH 7.66, whereas its living colonies continued to maintain the same net calcification rate under similar conditions. Recent studies on the Mediterranean red coral *Corallium rubrum* have contended that the shape of in hospite sclerites, grown under low pH conditions, significantly differed from that of the control [23], [28]. These results are in stark contrast to our findings and might be explained by the relatively thin tissue of *C. rubrum*, compared to the fleshy colonies of *O. macrospiculata*. The sclerites of *C. rubrum* are located near the colony surface, in contrast to those of *O. macrospiculata*, which are mostly embedded in the thick fleshy coenenchyme. It should be noted that the *O. macroscpicuta* sclerites demonstrated a repetitive pattern of damage, which was also quantified (this study: Figs. 3–5), whereas the studies on *C. rubrum* lack such data ([23]: Fig. S3; [28]: Figs. 3, 4). Nonetheless, a comparison between different octocoral species may further demonstrate the protective role played by the tissue as a barrier against such damage, and highlights the difference between organisms in the extant of their tissue protection. The results of our earlier study had revealed that several biological features of *O. macrospiculata* were not affected by the reduced seawater pH, thus suggesting the protective role of the living tissue [20]. Those findings are in congruence with the current ones, and further imply that the fleshy tissues of octocorals may act as a barrier that maintains a stable internal pH condition, thus preventing adverse effects on their internally-located sclerites. Undoubtedly, further studies are needed in order to elucidate the mechanisms of acid-based regulation in octocorals.

In the present study the reduced pH conditions led to a gradual corrosion of the isolated sclerites, with severity related to the pH level under the experimental conditions (Figs. 2, 3). It is suggested that the pits on the sclerites resulted from the dissolution and loss of corroded microscleres. Interestingly, the microscleres underwent a remarkably distinct repetitive dissolution pattern, observed at the microstructural level (Fig. 3). Stemmer et al. [29] presented SEM micrographs of *O. macrospiculata* sclerites, with microscleres similar to those found in our study (Fig. 3b, c). In that study the authors suggested that these pits might be a taxonomic characteristic of that species. Our study on the microstructure of *Ovabunda* sclerites [24] and examination of numerous colonies of the genus (unpublished data) do not show microscleres with pits, except after exposure to reduced pH conditions (this study). It might be that the Stemmer et al. [29] pits resulted from oxidation of the aldehyde group of the 7% formalin in which the colonies were fixed, which led to an acidic pH of the preservative. Harmful effects of formalin on the sclerites of a variety of octocoral museum specimens that have been preserved in formalin are frequently observed, ranging from a partial to even total dissolution (Y.B., personal observations). The dissolution mechanism that determines the unique microstructural changes of *Ovabunda* sclerites (Figs. 3, 4; [29]) under reduced pH conditions remains to be studied.

In summary, the current results demonstrate that the soft coral *O. macrospiculata* maintains the integrity of in hospite sclerites under reduced pH conditions, most probably due to the barrier supplied by its fleshy tissue. Therefore, we suggest that soft corals, in particular the zooxanthellate ones, are likely, at least on the experimental time scales of our study, to withstand the direct effects of ocean acidification, in terms of both their physiological performance [20] and skeletal integrity (this study). Determination of the time-scale for maintaining this skeletal robustness awaits further studies. In light of the competition required for space with the less resilient scleractinian corals, soft corals may thus have a significant advantage under above-normal acidic conditions.
