# Correction: Octocoral Tissue Provides Protection from Declining Oceanic pH

**DOI:** 10.1371/journal.pone.0102863

**Published:** 2014-07-10

**Authors:** The PLOS ONE Staff

The Figure Legend for Figure 3 is incorrect. The authors have provided a corrected version below.

**Figure 3:**
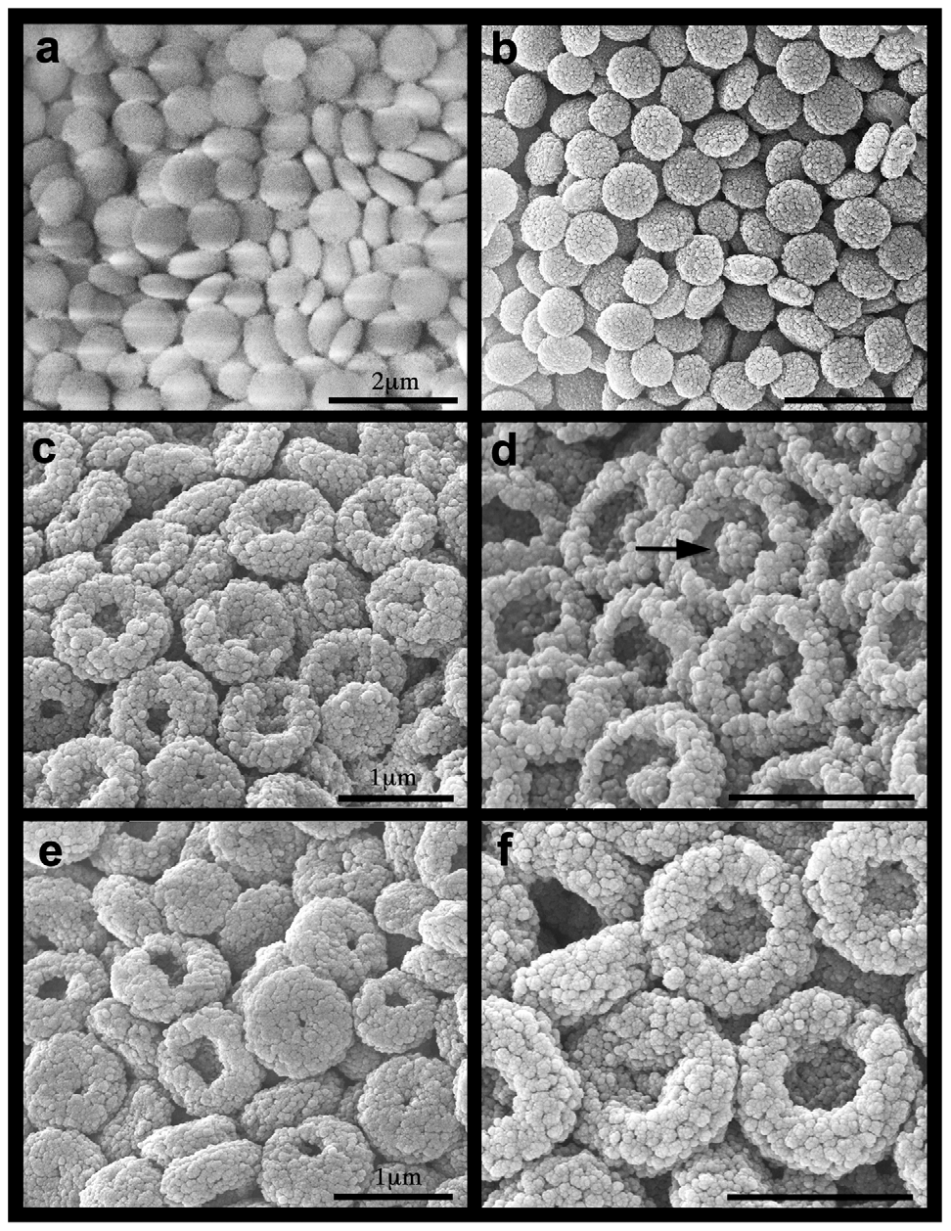
*Ovabunda macrospiculata*: ESEM images of isolated sclerites (December 2010 experiment). Day 31: a. pH 8.2 uncoated, b. gold coated, c, d. pH 7.6, e, f. pH 7.3. Arrow at d indicates dissolved circumferential zone. Scale at a applies also to b, scale at c applies also to d-f.
